# Multiparametric Cell Cycle Analysis Using the Operetta High-Content Imager and Harmony Software with PhenoLOGIC

**DOI:** 10.1371/journal.pone.0134306

**Published:** 2015-07-28

**Authors:** Andrew J. Massey

**Affiliations:** Vernalis Research, Granta Park, Cambridge, CB21 6GB, United Kingdom; St. Georges University of London, UNITED KINGDOM

## Abstract

High-content imaging is a powerful tool for determining cell phenotypes at the single cell level. Characterising the effect of small molecules on cell cycle distribution is important for understanding their mechanism of action especially in oncology drug discovery but also for understanding potential toxicology liabilities. Here, a high-throughput phenotypic assay utilising the PerkinElmer Operetta high-content imager and Harmony software to determine cell cycle distribution is described. PhenoLOGIC, a machine learning algorithm within Harmony software was employed to robustly separate single cells from cell clumps. DNA content, EdU incorporation and pHH3 (S10) expression levels were subsequently utilised to separate cells into the various phases of the cell cycle. The assay is amenable to multiplexing with an additional pharmacodynamic marker to assess cell cycle changes within a specific cellular sub-population. Using this approach, the cell cycle distribution of γH2AX positive nuclei was determined following treatment with DNA damaging agents. Likewise, the assay can be multiplexed with Ki67 to determine the fraction of quiescent cells and with BrdU dual labelling to determine S-phase duration. This methodology therefore provides a relatively cheap, quick and high-throughput phenotypic method for determining accurate cell cycle distribution for small molecule mechanism of action and drug toxicity studies.

## Introduction

The accurate determination of cell cycle perturbations is critically important in the development of small molecule and biological therapeutics especially those focused on novel treatments for cancer. Agents targeting the cell cycle machinery, DNA replication, mitosis, cell cycle checkpoints and oncogenic signalling are being or have been pursued. Understanding the mechanism of action of novel therapeutics in cancerous and non-cancerous cells is important for the progression of their development.

Traditionally, flow cytometry (FC) on ethanol fixed cells using propidium iodide to determine DNA content has been utilised to assign cells to specific phases of the cell cycle [1]. This approach has limitations namely an inability to separate G2 and M-phase cells, and a tendency to under estimate the S-phase population [2]. Multiparametric FC assays have been described that utilise DNA / BrdU / pHH3 (S10) or DNA / Ki67 / pHH3 (S10) content to accurately determine the fraction of cells in G1, S, G2 and M-phase of the cell cycle [3–5]. These assays, however, are still relatively low throughput and, for adherent cells, require additional manipulations such as trypsinisation that might affect the results.

High-content imaging is a plate based, automated fluorescence microscopy technique that allows the identification and quantification of cells based on their cellular phenotype and its use has become routine in toxicology and drug discovery [6–10]. Previous described methods using mulitparametric high content imaging to analyse cell cycle phases [11] do not describe robust methods for separating single cells from cell clumps. Here I describe a method to accurately separate single cells into cell cycle phase based on multiparametric marker expression using the Operetta high-content imager and Harmony software with PhenoLOGIC machine learning.

## Materials and Methods

### Cell lines and cell culture

All cell lines were purchased from the American Type Culture Collection (ATCC), established as a low passage cell bank and then routinely passaged in our laboratory for less than 3 months after resuscitation. HT29 and U87MG cells were routinely cultured in DMEM and SKOV-3 in McCoys 5a both containing 10% fetal calf serum (FCS) and 1% penicillin / streptomycin at 37°C in a normal humidified atmosphere supplemented with 5% CO_2_. For quiescence induction, cells were trypsinised and resuspended in media with 10% FCS, centrifuged and washed twice with FCS-free media and then resuspended in media containing 0.2% FCS and counted. Cells were subsequently plated in media containing 0.2% FCS and incubated for 72 hours before analysis.

### Chemicals

Compounds were purchased from the following suppliers and prepared as concentrated solutions in an appropriate solvent: camptothecin (C-3800) from LC Laboratories, gemcitabine (33275) from Apin Chemicals, oxaliplatin (2623) and carboplatin (2626) from Tocris, nocodazole (M-1404) from Sigma and etoposide (S1225), staurosporine (S1421), paclitaxel (S1150), doxorubicin (S1208) and VX-680 (S1048) from Selleckchem.

### High-content cell cycle analysis

10 000 cells were plated per well of a CellCarrier 96 well plate (PerkinElmer) and allowed to attach for 24 hours. Cells were labelled with 10μM EdU for 30 minutes immediately prior to fixation with 3.7% paraformaldehyde in PBS at room temperature for 15 minutes. Cells were washed twice in PBS then twice in 3% BSA in PBS before permeabilisation with 0.5% Triton X100 in PBS for 20 minutes at room temperature. Cells were washed twice with 3% BSA in PBS before incorporated EdU was labeled with AF488 using a Click-iT EdU labeling kit (C10337, LifeTechnologies). 50μl of Click-iT reaction cocktail was used per well and consisted of 43μl Click-iT reaction buffer, 2μl copper protectant, 0.12μl AF488 picolyl azide and 5μl reaction buffer additive. Following blocking for 30 minutes with 5% normal goat serum in PBS, cells were incubated with an anti-pHH3 (S10) primary antibody (mouse: #9706, Cell Signaling Technologies, 1:400 or rabbit: #3377, Cell Signaling Technologies, 1:1600) diluted in antibody dilution buffer (1% BSA, 0.3% Triton X100 in PBS) at 4°C for 16 hours. For Ki67 detection, cells were incubated with primary antibody (rabbit: #9129, Cell Signaling Technologies, 1:400) diluted in antibody dilution buffer at the same time as pHH3 (S10) detection. Cells were washed with PBS then incubated with an anti-mouse AF546 and/or anti-rabbit AF647 labelled secondary antibody (1:500, LifeTechnologies) and Hoechst 33342 (1μg/ml) in antibody dilution buffer at room temperature for 60 minutes. Following washing with PBS, cells were imaged with an Operetta high-content imaging system (PerkinElmer) at 10x magnification.

### Determination of S-phase duration using EdU and BrdU dual pulse labelling

10 000 HT29 cells were plated per well of a CellCarrier 96 well plate (PerkinElmer) and allowed to attach for 24 hours. Cells were labelled with 10μM EdU for 30 minutes, media removed and cell incubated for 120 minutes before being labelled with 20μM BrdU (Abcam) for 30 minutes. Cells were fixed with 3.7% paraformaldehyde in PBS at room temperature for 15 minutes then washed three times with PBS before permeabilisation with 0.5% Triton X100 in PBS for 20 minutes at room temperature. Following three washes in PBS, DNA was denatured using 4M HCl and incubation at room temperature for 10 minutes. Cells were again washed twice with PBS then twice with 3% BSA in PBS before EdU incorporation was determined using click chemistry and an AF647 picolyl azide as described above. BrdU incorporation was determined by incubation with the monoclonal antibody MoBU-1 (LifeTechnologies, B35128, 1:200) at +4°C overnight. Following washing, this was subsequently detected using an anti-mouse AF488 labelled secondary antibody (1:500, LifeTechnologies) and Hoechst 33342 (1μg/ml).

### High-content analysis of RNA levels

10 000 cells were plated per well of a CellCarrier 96 well plate (PerkinElmer) and allowed to attach for 24 hours. Cells were fixed with 100% methanol (-20°C) for 10 minutes then washed three times with PBS. Cells were subsequently stained with 200μl Hoechst 33342 (2.5μg/ml) in PBS for 30 minutes at room temperature then 5μl of Pyronin Y (Sigma, 82μg/ml) or 5μl of RNAselect (LifeTechnologies, 20.5μM) added and further incubated at room temperature for 20 minutes. Following washing with PBS, cells were imaged with an Operetta high-content imaging system at 20x magnification.

### Analysis sequence

Images were analysed using Harmony software with PhenoLOGIC (PerkinElmer) with the following analysis sequence. Building blocks are defined with [] and adjustable parameters in italics.

1. [Input Image]–*Individual Planes*, *FFC Basic*
2. [Find Nuclei]–Channel: *HOECHST 33342*; Method: *B*; Output Population: *Nuclei*
3. [Select Population]–Population: *Nuclei*; Method: *Common Filters (Remove Border Objects)*; Output Population: *Nuclei Selected*
4. [Calculate Morphology Properties]–Population: *Nuclei Selected*; Region: *Nucleus*; Method: *Standard (Area*, *Roundness)*
5. [Calculate Morphology Properties (2)]–Population: *Nuclei Selected*; Region: *Nucleus*; Method: *STAR (HOECHST 33342*, *Symmetry*, *Threshold Compactness*, *Axial)*
6. [Calculate Intensity Properties]–Channel: *HOECHST 33342*; Population: *Nuclei Selected*; Region: *Nucleus*; Method: *Standard (Sum)*; Output Properties: *DNA*
7. [Calculate Intensity Properties (2)]–Channel: *AF 488*; Population: *Nuclei Selected*; Region: *Nucleus*; Method: *Standard (Sum)*; Output Properties: *EdU*
8. [Calculate Intensity Properties (3)]–Channel: *AF 546*; Population: *Nuclei Selected*; Region: *Nucleus*; Method: *Standard (Mean)*; Output Properties: *pHH3 (S10)*
9. [Select Population (2)]–Population: *Nuclei Selected*; Method: *Linear Classifier*; Output Population A: *Singlets*; Output Population B: *Multiplets*
10. [Calculate Properties]–Population: *Singlets*; Method: *By Formula (Formula*: *log10(a)*, *Variable A*: *EdU Sum)*; Output Property: *Log EdU*
11. [Calculate Properties]–Population: *Singlets*; Method: *By Formula (Formula*: *a/#value*, *Variable A*: *DNA Sum)*; Output Property: *Normalised DNA* where <*#value*> refers to the estimated G1 peak value.
12. [Select Population (3)]–Population: *Singlets*; Method: *Filter by Property (values for Normalised DNA*, *Log EdU and pHH3 (S10) Mean entered for particular cell cycle phase)*
13. [Select Population (x)]–*repeat above step for all cell cycle phases*
14. [Define Results]

### Data analysis

DNA histograms and scatter plots were created from 5 images per well and equated to between 3000 and 6000 cell events. DNA histograms were generated by binning the normalised DNA (output from analysis step (11) above) into 300 equal sized bins using Microsoft Excel.

## Results and Discussion

### Quantification of cell cycle phase based on DNA content analysis

**Fig 1:**
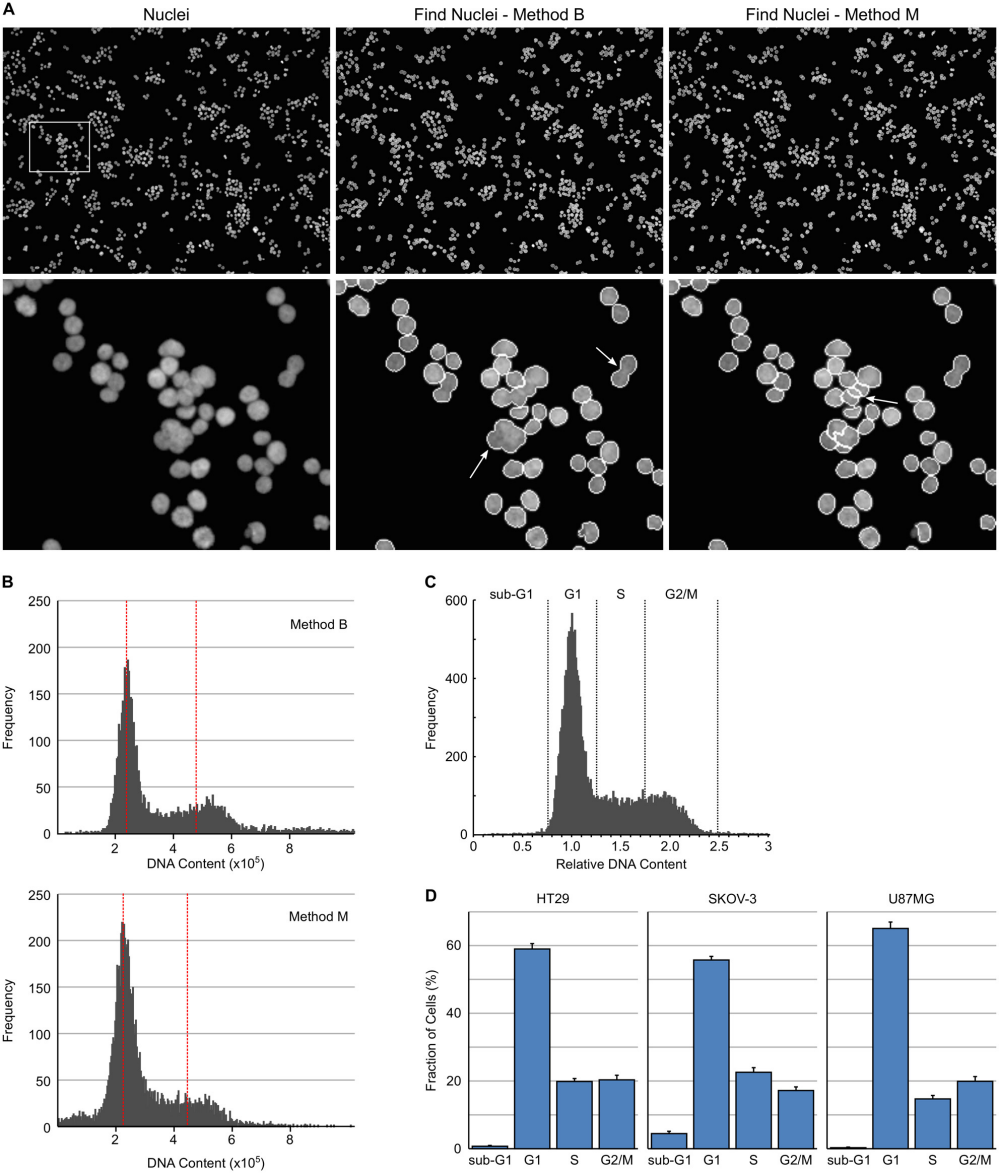
High-content cell cycle analysis based on DNA content. (A) Example images of HT29 cells with nuclei stained with Hoechst 33342 and then segmented with Harmony software using Find Nuclei Method B or Method M. Arrows indicate examples of incompletely segmented nuclei. (B) DNA content histograms of Hoechst 33342 stained HT29 cells, prior to application of the PhenoLOGIC algorithm, using Find Nuclei method B and M. Red dotted lines indicate the position of the G1 and G2 peak. (C) DNA content histograms obtained from HT29 cells showing gating to identify cell cycle distribution. (D) Cell cycle distribution for asynchronous HT29, SKOV-3 and U87MG cells determined from DNA content histograms. Values are the average of 8 technical replicates ± SD.

The measurement of nuclear DNA content and its use to assign cells to a particular phase of the cell cycle is one of the most wide spread applications of flow cytometry. All of the established methods use dyes that specifically and stochastically bind nucleic acids and whose fluorescence is enhanced upon binding. The same approach and principles can be applied to determine cell cycle phase with a high-content imaging system [12]. Asynchronous HT29 cells growing in normal cell culture media were fixed with paraformaldehyde and subsequently stained with Hoechst 33342. Images were collected using a 10x long working distance objective with 5 fields per well captured. An example of a collected image is shown in Fig 1A. The images were first analysed using Harmony software with building blocks 1–6, 9 and 14 of the analysis sequence described in materials and methods. The [Define Results] building block was set to report Singlets: DNA Sum: Mean with Single Cell Results: Selected.

Optimal segmentation of nuclei and separation of single cells from clumped cells is critical for accurate cell cycle determination. The segmentation of nuclei by two different [Find Nuclei] methods (B and M) was compared. Method M produced greater splitting of nuclei than method B (Fig 1A) but had a tendency to separate two overlapping nuclei into three nuclei. Example DNA histograms of the same HT29 cells using [Find Nuclei] methods B and M are illustrated in Fig 1B. As can be seen, method M increases the number of cells with a DNA content less than G1 compared to method B. Method B was therefore selected as the nuclei segmentation method of choice. In flow cytometry, single cells are separated from clumps by gating on a plot of DNA area versus DNA width or peak. Using similar plots in Harmony did not allow the reliable separation of single nuclei from multiple nuclei (S1 Fig). PhenoLOGIC is a machine-learning algorithm in Harmony software and was therefore used to separate the two phenotypes. It is based on a linear classifier and can be trained to enable the high-content software to recognise and classify cells based on phenotype. Building block 9 ([Select Population (2)]) was used to train two classes of cells—class A: Singlets and class B: Multiplets using approximately 150 cells from each class. The linear classifier generated from this analysis used morphological properties to separate the two cell populations namely, nucleus axial length ratio, nucleus symmetry, nucleus axial small length and nucleus roundness with a “goodness” of 2.24. The “goodness” value indicates the quality of the separation but does not provide information about the distribution of classification results. This approach worked well for cells that have a tendency to grow as clumps such as HT29 and U87MG cells. For cells that grow as a more uniform monolayer (such as SKOV-3 cells) finding sufficient class B cells to train the linear classifier required the analysis of multiple wells and fields. In general, the linear classifier only required training when applied to a new cell line. However, if a compound induces significant changes to nuclear morphology, additional re-training using examples of treated cells may be necessary to ensure accurate cell separation. Using PhenoLOGIC to separate the cell populations had the added advantage of also filtering out any fluorescent debris from subsequent analyses.

From the single cell results, a histogram of DNA Sum was generated using additional software such as Excel, Vortex or SpotFire and the intensity corresponding to the centre of the G1 peak determined. This value is fed back into building block 11 of the analysis sequence to replace *#value* in the formula *a/#value*. This results in a DNA histogram of normalised DNA (Fig 1C) which can be used to categorise individual cells according to their DNA content in the same way as a DNA histogram is interrogated by flow cytometry. The normalised DNA content parameter was determined from untreated control wells on a per-plate basis and then applied to all wells on that plate. Multiples of building block 12 can be generated to classify the cells based on normalised DNA: <0.75, sub G1; 0.75–1.25, G1; 1.25–1.75, S; 1.75–2.5, G2/M; >2.5, >G2/M. This methodology was used to determine the cell cycle distribution of asynchronous HT29, SKOV-3 and U87MG cancer cell lines growing in normal media (Fig 1D).

### Multiparametric analysis of cell cycle phase

Cell cycle classification based on DNA content alone has limitations namely an inability to separate the G2 and M cell populations and a tendency to underestimate the S-phase population. A multiparametric high-content assay was therefore developed that measures DNA content using Hoechst 33342 dye staining, incorporation of EdU to measure cells actively synthesising DNA [13] and pHH3 (S10) expression to determine mitotic cells [14, 15]. Asynchronous HT29 cells were pulse labelled with 10μM EdU for 30 minutes to label cells actively synthesising DNA before being fixed with paraformaldehyde. EdU incorporation was determined using a Click-iT EdU AF 488 labelling kit and pHH3 (S10) (whose expression correlates closely with DNA condensation in mitosis) with either a mouse or rabbit monoclonal antibody and AF546 or AF647 labelled secondary antibody. Images were collected using a 10x long working distance objective with 5 fields per well captured.

The images were analysed as per the DNA only method described above with the addition of some extra analysis building blocks. These include two additional [Calculate Intensity Properties] building blocks to determine EdU Sum and pHH3 (S10) Mean whilst the [Define Results] building block was set to report Singlets: DNA Sum: Mean, Singlets: EdU Sum: Mean, and Singlets: pHH3 (S10) Mean: Mean, with Single Cell Results: Selected. When training the PhenoLOGIC linear classifier, including pHH3 (S10) positive single cells in the training set to include pHH3 (S10) mean intensity as part of the classifier can improve the detection of mitotic cells with some cell lines.

**Fig 2:**
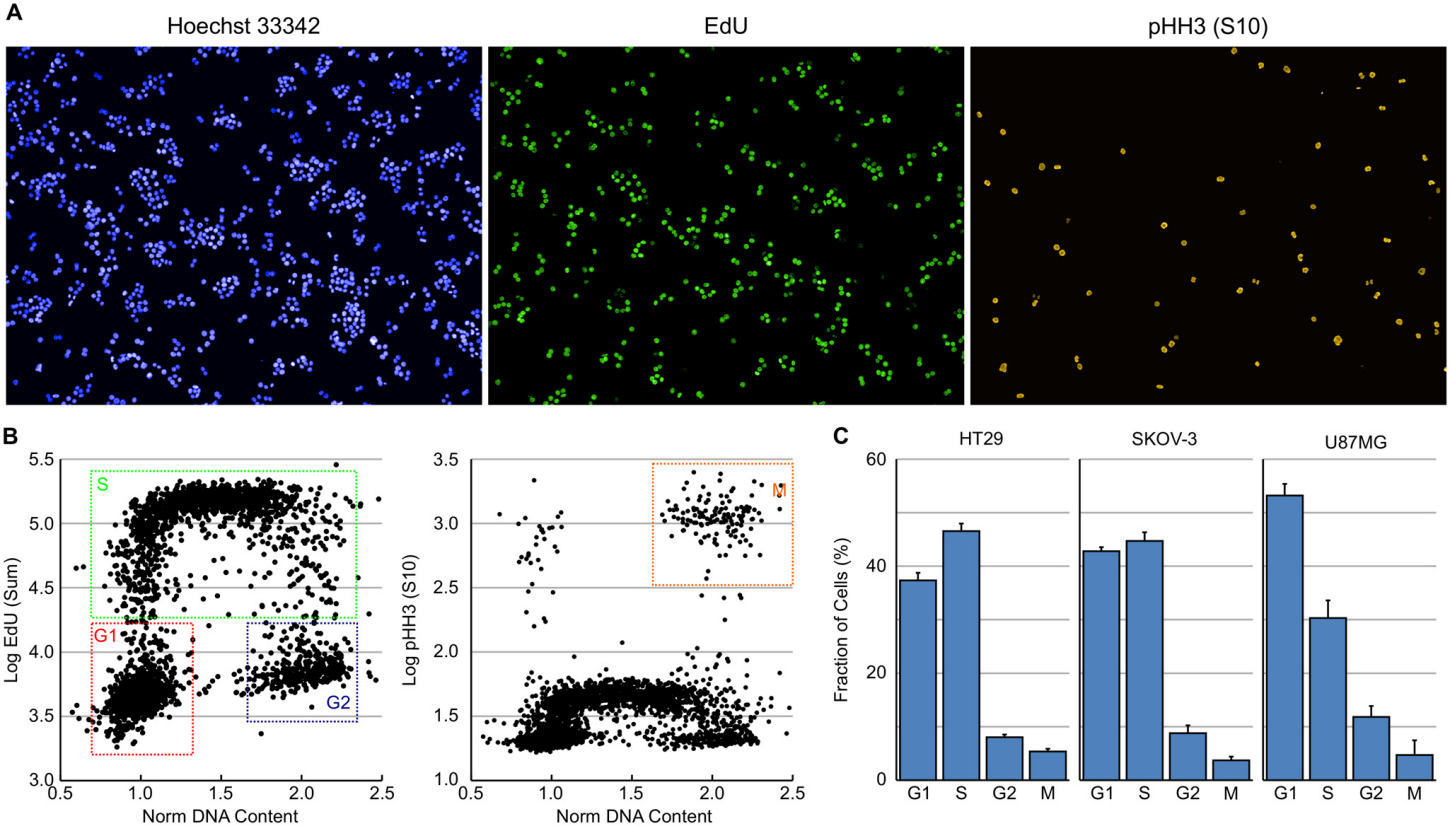
Multiparametric analysis of cell cycle distribution. (A) Example images demonstrating DNA staining with Hoechst 33342, EdU incorporation and pHH3 (S10) expression in untreated HT29 cells. (B) Plots of DNA content versus log EdU content and DNA content versus log mean pHH3 (S10) expression to determine the major cell cycle phases in asynchronous HT29 cells. (C) Cell cycle distribution for asynchronous HT29, SKOV-3 and U87MG cells determined from multiparametric high-content images. Values are the average of 8 technical replicates ± SD.

From the single cell results, the DNA content was normalised as before and the EdU Sum logged to simplify the setting of the necessary gates. Plots of Normalised DNA versus Log EdU Sum and Normalised DNA versus pHH3 (S10) Mean can be subsequently used to set gates to classify cells into sub-G1, G1, S, G2, M and >G2/M populations using the [Select Population] building block. For example in the case of HT29 cells detailed in Fig 2A, the G1 population is defined by Normalised DNA 0.7–1.4, Log EdU Sum 3.2–4.3 and pHH3 (S10) Mean <300 (Fig 2B). This methodology was used to determine the G1, S, G2 and M populations of asynchronous HT29, SKOV-3 and U87MG cancer cell lines growing in normal media (Fig 2C). As can be seen from the data in Fig 2C, the fraction of S-phase cells is greater and the fraction of G1-phase cells lower than when just DNA is used to determine the cell cycle phase (Fig 1D). Likewise, the fraction of mitotic cells is clearly separated from the G2 population.

**Fig 3:**
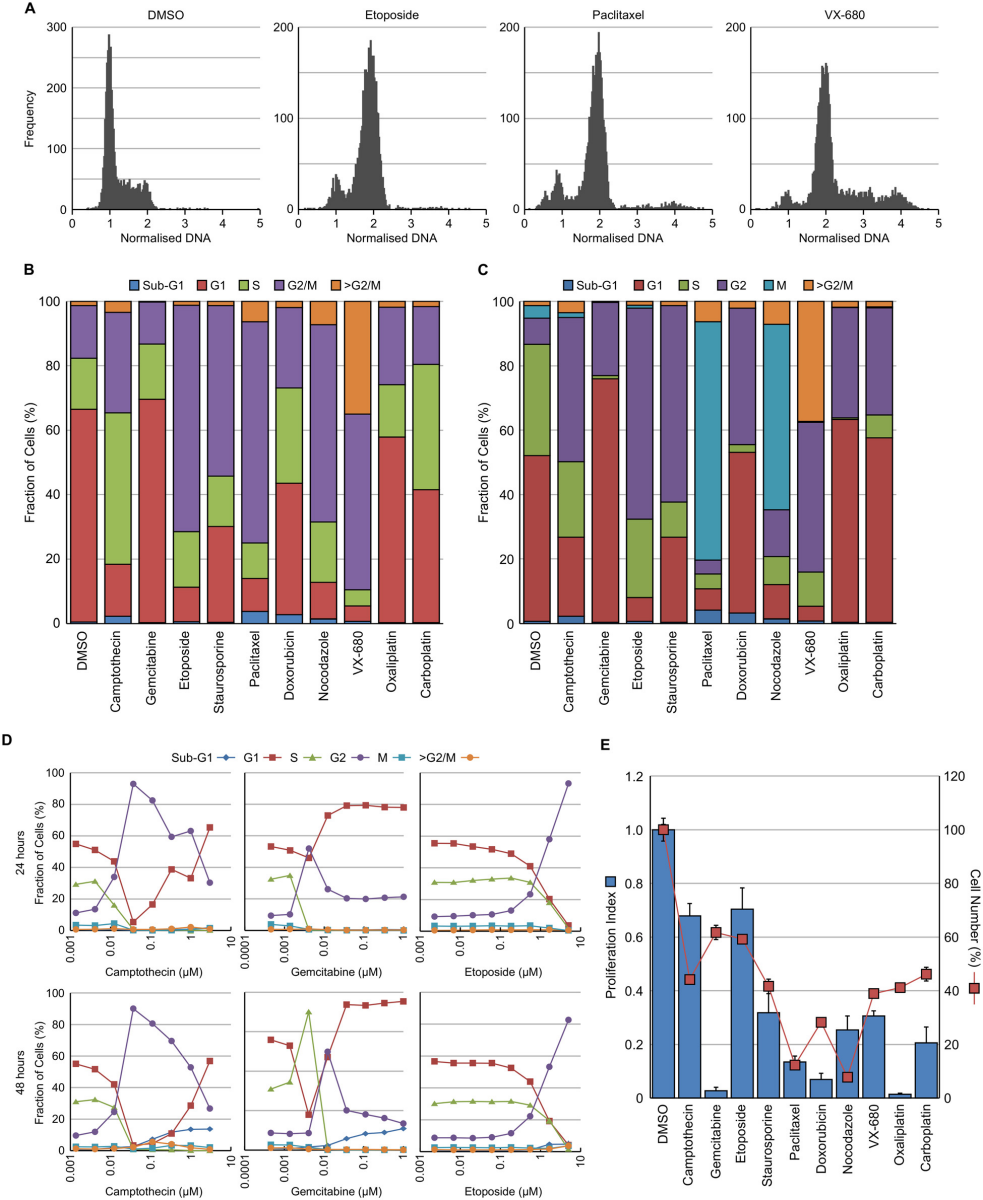
Validation of image analysis protocol. HT29 cells were treated with the indicated compound for 24 hour and then fixed and stained with Hoechst 33342, EdU and pHH3 (S10). (A) Example histograms following treatment with DMSO, etoposide, paclitaxel or VX680. Cell cycle distribution was determined using either DNA histogram analysis (B) or multiparametric analysis (C). (D) Time course and dose response of cell cycle changes determined using the multiparametric method for cells treated with camptothecin, gemcitabine or etoposide. (E) Proliferation index (EdU positive cells treated / EdU positive cells control) and cell number were determined from the images. Values are the average of 6 technical replicates.

To validate the image analysis methodology and ensure that accurate cell cycle changes could be detected by this multiparametric approach, HT29 cells were cultured with a range of agents known to induce cell cycle perturbations for 24 hours. These agents, their mechanism of action and their predicted cell cycle response are summarised in Table 1. Example DNA histograms for DMSO treated control cells plus cells treated with etoposide, paclitaxel or VX-680 [16] are shown in Fig 3A. The methodology described above was used to determine the cell cycle distribution based solely on DNA content (Fig 3B) or on DNA, EdU and pHH3 (S10) levels (Fig 3C). Both methods reliably determined changes in cell cycle distribution following treatment with a wide variety of agents known to perturb the cell cycle. The multiparametric method has a distinct advantage in being able to separate the G2 and M populations and this was obvious when comparing the cell cycle response between, for example, etoposide which induced G2 arrest and paclitaxel which induced mitotic arrest. The multiparametric method was further validated by determining the cell cycle changes induced following 24 or 48 hour treatment with decreasing concentrations of camptothecin, gemcitabine or etoposide. The method reliably detected dose dependent changes in cell cycle responses to all three agents that were robust at both time points (Fig 3D).

**Table 1:** Agents used to validate image analysis methodology.

Agent	Conc (μM)	Target / MOA	Predicted Cell Cycle Arrest
Camptothecin	1	Topoisomerase I	G2
Gemcitabine	0.2	Antimetabolite	G1, G2
Etoposide	5	Topoisomerase II	G2
Staurosporine	0.2	Pan-kinase	Sub-G1, G2
Paclitaxel	0.2	Microtubule Stabilisation	M
Doxorubicin	3	Topoisomerase II	G2
Nocodazole	0.3	Microtubule Destabilisation	M
VX-680	0.4	Aurora B	4N, 8N
Oxaliplatin	100	DNA Alkylation	G1, G2
Carboplatin	500	DNA Alkylation	G1, G2

Cell proliferation could also be determined using the multiparametric approach by determining the number of cells actively undergoing DNA synthesis (EdU positive). This was, in turn, compared to the number of cells (Fig 3E). For example, 24 hour treatment with gemcitabine completely inhibits DNA synthesis but only reduces cell number by around 40% compared to the DMSO control. In comparison, camptothecin did not inhibit cell proliferation as dramatically as gemcitabine but had a greater effect on cell survival. This provides additional information on the mechanism of drug action without the requirement of additional experiments.

Estimating cell cycle distribution based on DNA content alone is a relatively rapid and low cost method allowing the rapid screening and evaluation of numerous compounds. However, including an analysis of DNA synthesis (EdU) and a marker of mitosis allowed the separation of cells arrested in G2 from those in mitosis and generated extra data on cell proliferation. Another advantage of this method is that it can be coupled with an additional pharmacodynamic (PD) biomarker to determine the cell cycle distribution of biomarker negative or positive cells for example following compound treatment or under different growth conditions. A drawback of using EdU (or BrdU) to label S-phase cells is that it will only label and therefore be detected in cells undergoing active DNA replication. Drugs that block DNA synthesis (such as gemcitabine or hydroxyurea) may appear to be blocked in G1 and/or G2 as they stain negatively for EdU but are in fact blocked in S. In these cases, interrogating the DNA histogram or staining the cells for an additional marker such as cyclin A, PCNA, geminin or Ki-67 will provide extra information on S-phase effects. The three Alexa dyes selected (AF488, AF546 and AF647) are compatible with the standard filter set of the Operetta. By having a choice of pHH3 (S10) antibody (rabbit or mouse) allows the assay to be coupled with mouse or rabbit PD marker antibodies. Three potential applications of the flexible assay were further evaluated: identification of the cell cycle phase of γH2AX positive cells following treatment with DNA damaging cytotoxic drug, the determination of S-phase duration using a EdU/BrdU double staining approach and the determination of the fraction of cells in G0 based on Ki67 expression.

### Identification of cell cycle phase of γH2AX positive cells following treatment with a DNA damaging cytotoxic drug

DNA damaging cytotoxic chemotherapeutic agents and ionizing radiation are the mainstay of current cancer treatment regimens. These agents target the DNA in cancer cells and induce DNA damage either directly through DNA adduct formation (for example cisplatin) or indirectly via inhibition of DNA synthesis (for example gemcitabine) or DNA unwinding (for example etoposide). Following DNA damage, the histone variant H2AX is rapidly phosphorylated on serine 139 (γH2AX) at sites of DNA damage and is required for the subsequent recruitment of components of the various repair pathways. γH2AX is a recognised biomarker for DNA double strand breaks [17–19].

**Fig 4:**
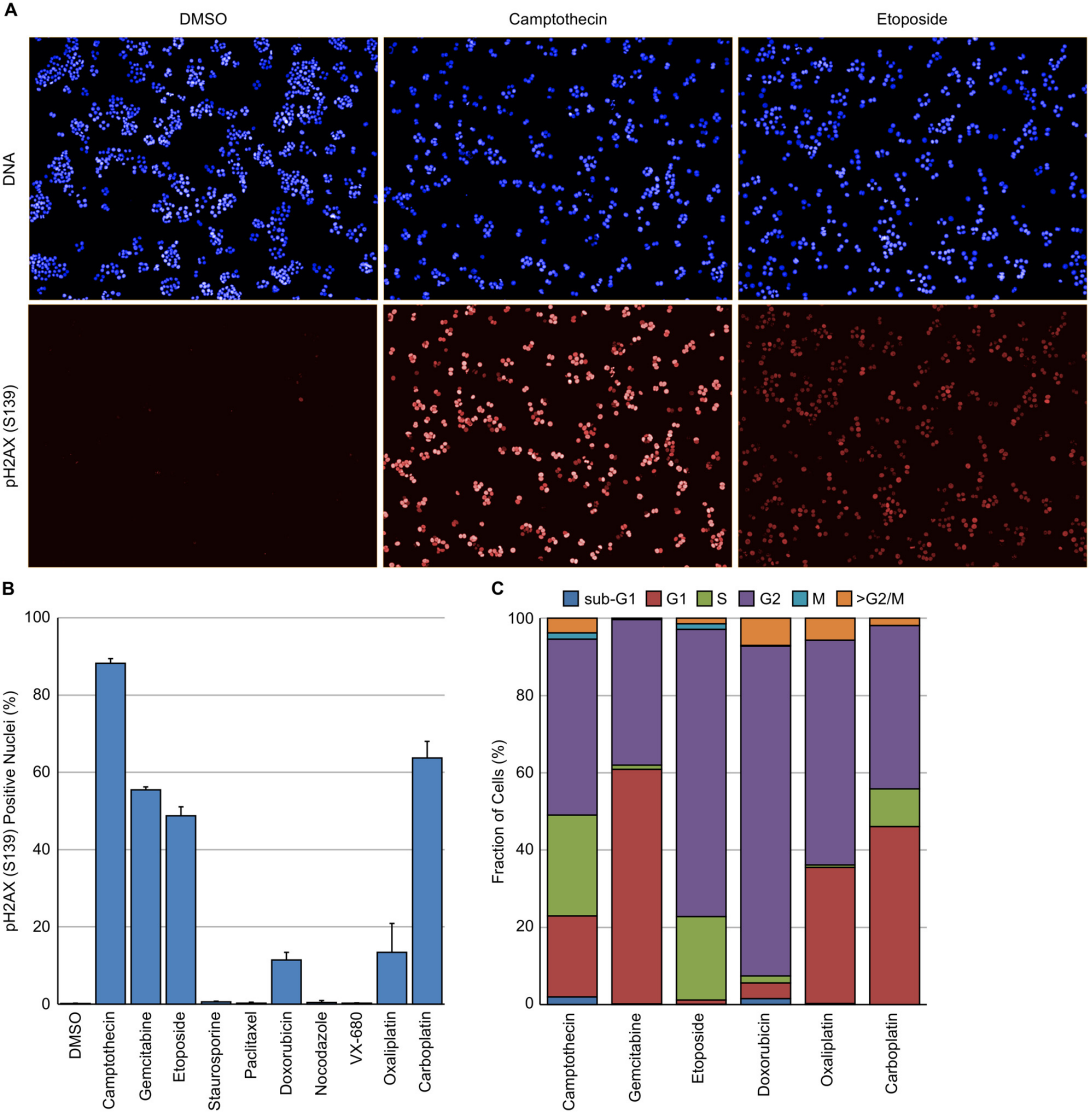
Determination of cell cycle phase of γH2AX positive cells following treatment with a cytotoxic DNA damaging agent. HT29 cells were treated with the indicated compound for 24 hour and then fixed and stained with Hoechst 33342, EdU, pHH3 (S10) and pH2AX (S139). (A) Example images from HT29 cells treated with DMSO, camptothecin or etoposide demonstrating the presence of pH2AX (S139) positive (γH2AX) nuclei. (B) Quantification of the fraction of γH2AX positive nuclei following treatment with the various agents. (C) Cell cycle distribution of γH2AX positive nuclei following multiparametric analysis. Values are the average of 6 technical replicates ± SD.

HT29 cells were treated for 24 hours with the agents indicated in Table 1 and subsequently stained for DNA, EdU, pHH3 (S10) and pH2AX (S139). Camptothecin, gemcitabine, etoposide, doxorubicin, oxaliplatin and carboplatin all induced a clear increase in nuclear localised γH2AX (Fig 4A and 4B). The cell cycle distribution of the γH2AX positive nuclei following treatment with these agents was subsequently determined (Fig 4C). As can be seen, the cell cycle distribution of the γH2AX positive cells was dependent on the agent with significant differences between agents. For example, etoposide and doxorubicin induced DNA damage in predominantly G2 cells whilst camptothecin induced DNA damage in cells in G1, S and G2. Very little DNA damage, as measured by γH2AX expression, was observed in mitotic cells. These differences are most likely reflective of the molecular mechanism of action of these agents.

### Determination of S-phase duration by EdU / BrdU dual pulse labelling

Sequential labelling of cells with halogenated thymidine analogues has been routinely used to determine additional information on cell cycle kinetics [20, 21]. The availability of a mouse monoclonal antibody, MoBU-1, that specifically recognises BrdU and does not cross react with EdU allows dual-pulse labelling with EdU and BrdU [22, 23]. This dual-pulse labelling approach can be utilised to estimate, among other things, the S-phase duration.

**Fig 5:**
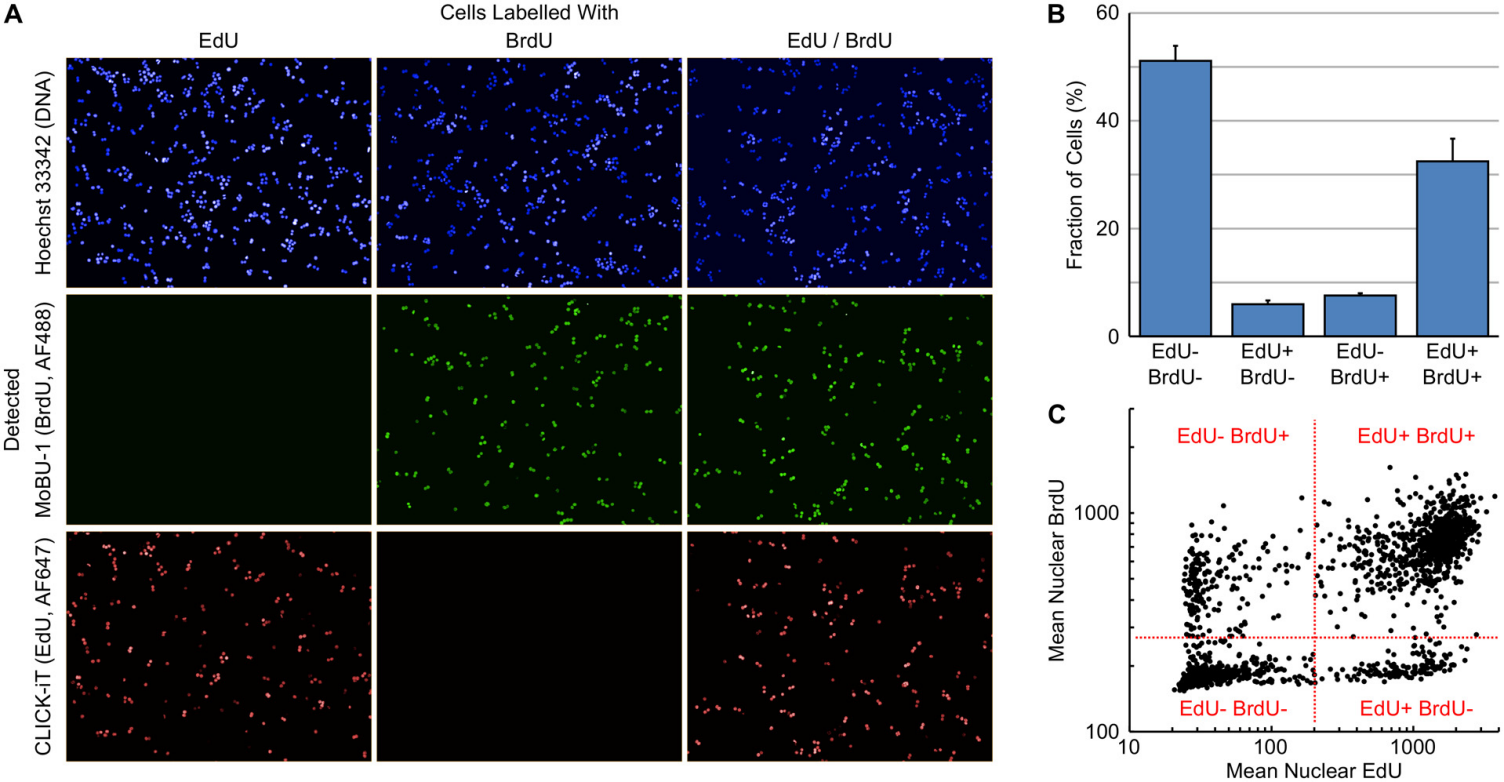
Estimation of S-phase Duration using EdU-BrdU Dual Pulse Labelling. (A) Images of HT29 cells labelled with various combinations of EdU and BrdU demonstrating the selectivity of Click-iT chemistry for EdU and MoBU-1 antibody for BrdU. (B) Percentage of cells labelled with the various combinations of EdU and BrdU. Values are the average of 6 replicates ± SD. (C) Dot plot of Mean Nuclear EdU versus Mean Nuclear BrdU demonstrating the positioning of gates for the various cell populations.

The rate of progression through S-phase can be estimated from the number of cells exiting S-phase in a given unit of time. To determine this, HT29 cells were sequentially labelled with EdU for 30 minutes, EdU containing media removed, incubated in label free media for 120 minutes then labelled with BrdU for 30 minutes. It is also possible to sequentially label the cells without washing out the first pulse. However, the order of nucleotide addition is important as BrdU is preferentially incorporated into DNA over EdU; labelling in this order means that the removal of EdU at the end of the first labelling period before the addition of BrdU is not necessary. Incorporated EdU was detected using click chemistry and BrdU with the monoclonal antibody MoBU-1. Fig 5A demonstrates the lack of cross reactivity between the Click-iT labelling reaction and BrdU, and the antibody MoBU-1 and EdU. S-phase duration (T_s_) was estimated using the formula: T_s_ = t/ΔS where t is the time between labelling and ΔS is the fraction of cells that have left S-phase during time t. ΔS is calculated as Cells_EdU+ BrdU-_ / Cells_EdU+ BrdU+_. The fraction of EdU+ BrdU+ and EdU+ BrdU- cells (Fig 5B) was rapidly determined by gating on Mean Nuclear EdU and Mean Nuclear BrdU intensities (Fig 5C). For HT29 cells this resulted in a T_s_ of 10.9 ± 1.1 hours (n = 6). From this, an approximate doubling time can be estimated using the formula T_p_ = k.100.T_s_/% Cells_BrdU+_ (where k = 0.8 for cells with a T_p_ < 50 hours and 0.7 for cells with a T_p_ > 50 hours). For the HT29 cells in this experiment, this resulted in a T_p_ of 20.5 ± 2.2 hours (n = 6) which correlates closely with the ATCC estimated doubling time of 23 hours.

### Quantification of the G0 phase using RNA content or Ki67 expression

The G0 phase of the cell cycle defines cells which are not actively dividing or preparing to divide (often referred to as quiescence) and is usually induced by a lack of growth factors or nutrients. This phase of the cell cycle is generally accepted to be reversible with cells re-entering the G1-phase of the cell cycle when conditions again become permissive of cell division. There is a strong correlation between quiescence and ribosome biogenesis with serum starvation inducing a decrease in rRNA synthesis and ribosome biogenesis [24]. Quantification of cellular DNA and RNA content by FC following Hoechst 33342 and Pyronin Y staining has been used to quantify the G0 phase of the cell cycle [1]. DNA staining with Hoechst 33342 followed by RNA staining with Pyronin Y or the RNA selective dye, RNAselect, to determine the fraction of G0 cells was evaluated by high-content imaging.

**Fig 6:**
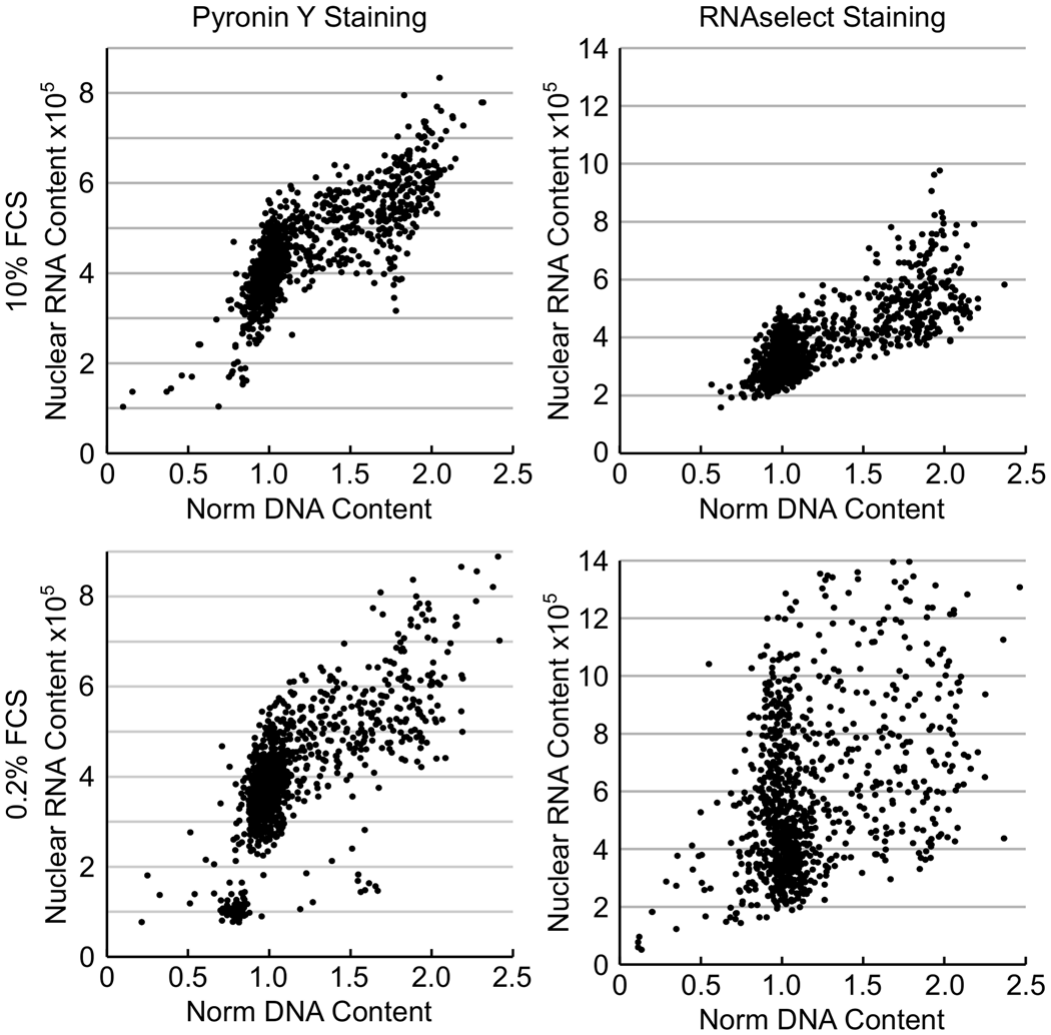
Determination of cellular quiescence using nuclear RNA staining. HT29 cells growing in 10% FCS or in 0.2% FCS for 72 hours were fixed and stained with Pyronin Y or RNAselect for determination of RNA and total DNA content. Plots of total nuclear DNA content versus total nuclear RNA content.

HT29 cells growing in either 10% or 0.2% FCS were fixed with cold methanol before being stained with Hoechst 33342 for 30 minutes then Pyronin Y or RNAselect for 20 minutes. Sequential staining is critical as Pyronin Y can also bind DNA and the prestaining of DNA with Hoechst 33342 blocks this. Cells were imaged with the Operetta using a 20x long working distance objective, 5-fields well with Pyronin Y detected using the AF546 channel and RNAselect the AF488 channel. Total nuclear DNA and total nuclear RNA were quantified and plotted (Fig 6). As can be seen, Pyronin Y and RNAselect staining and quantification gave contradictory results. Decreasing the concentration of FCS increased the fraction of cells with reduced Pyronin Y fluorescence (as would be predicted) but increased the fraction of cells with increased RNAselect staining. Similar results were obtained with SKOV-3 and U87MG cells. The reason for the contradictory results obtained with these two dyes was not apparent. However, a potential explanation may be due to differences in their selectivity for RNA versus DNA (RNAselect is more selective than Pyronin Y) and there may be differences in the preference for the kind of RNA they bind to / sub-cellular compartment where they accumulate. High background staining was observed with Pyronin Y resulting in a poor signal: noise ratio which may also contribute to the differences observed. An alternative approach to identify and quantify the G0 population was therefore evaluated.

**Fig 7:**
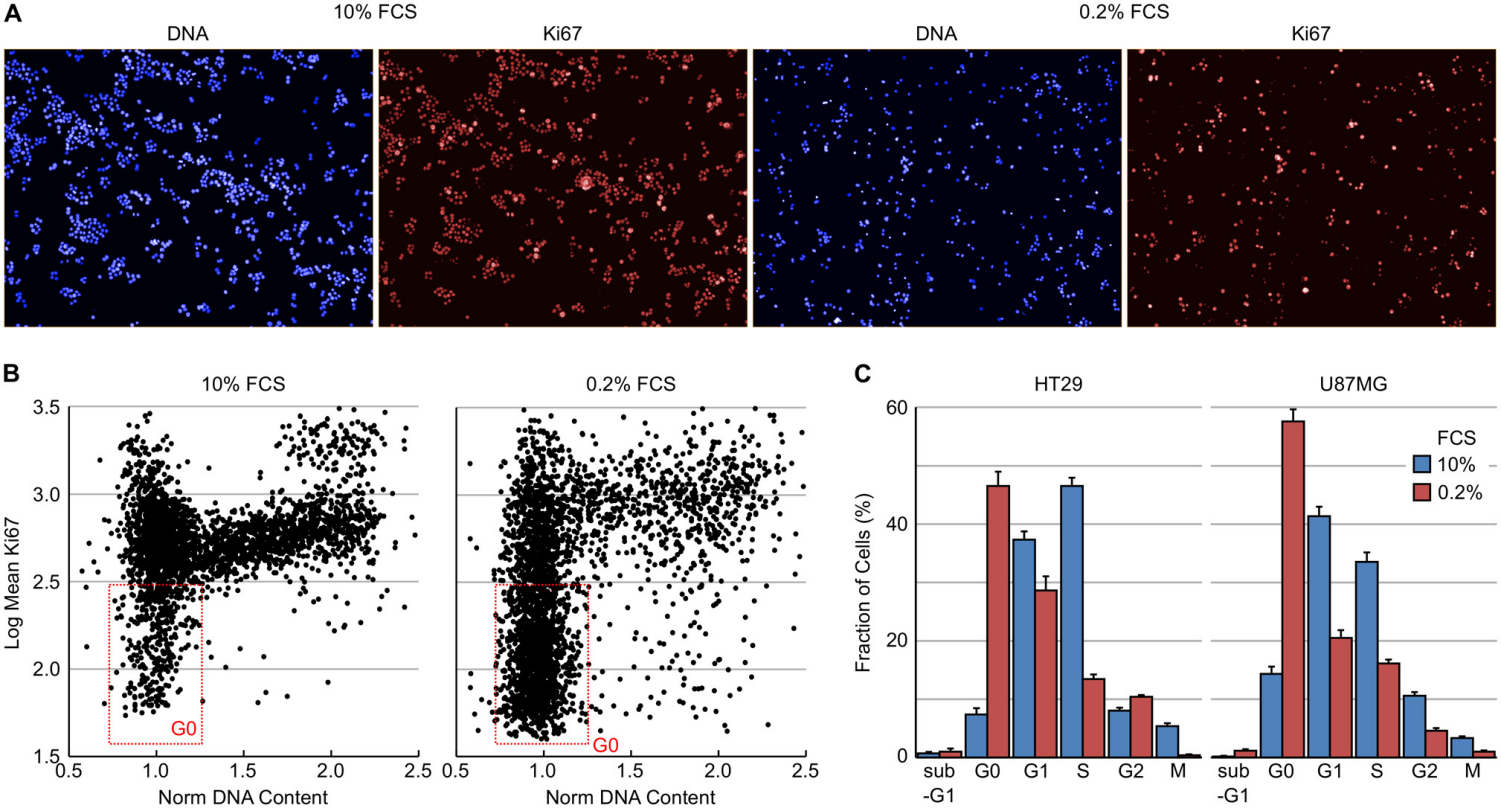
Determination of cellular quiescence using nuclear Ki67 expression levels. HT29 cells growing in 10% FCS or in 0.2% FCS for 72 hours were fixed and stained for Ki67 and pHH3 (S10) expression, total EdU and total DNA content. (A) Example images demonstrating Ki67 staining in HT29 cells. (B) Plots of total DNA versus log mean Ki67 fluorescence intensity demonstrating the identification of the quiescent (Ki67 negative) cell population. (C) Cell cycle distribution of HT29 and U87MG cells grown in 10% or 0.2% FCS determined from multiparametric high-content images. Values are the average of 8 technical replicates ± SD.

Ki67 is a nuclear non-histone protein that is universally expressed in proliferating cells (including those in G1, S, G2 and M) but not quiescent (G0) cells and is often used as a biomarker of cell proliferation rate in tumour biopsies [25–27]. The multiparametric cell cycle method described above was utilised to determine the fraction of cells in G0 by immunostaining for Ki67 expression (using a rabbit antibody and an AF647 labelled secondary antibody) in combination with DNA and EdU content analysis, and pHH3 (S10) expression levels (using a mouse antibody and an AF546 labelled secondary antibody). Example images of HT29 cells are shown in Fig 7A. From a plot of normalised DNA content versus log mean Ki67 intensity of HT29 cells grown in 10% or 0.2% FCS it was possible to identify the fraction of quiescent cells (Fig 7B). Plotting the log Ki67 mean allows better visualisation of the range of Ki67 intensities. The [Select Population] building block can therefore define the G0 population as Normalised DNA 0.7–1.4, Log EdU Sum 3.2–4.3, pHH3 (S10) Mean <300 and Ki67 Mean <200. Serum starvation of HT29 or U87MG induced a dramatic increase in the fraction of Ki67 cells in both cell lines (Fig 7C). In HT29 cells, serum starvation increased the G0 fraction from 7.4 to 46.5%. Likewise, serum starvation induced a large decrease in the fraction of cells actively undergoing DNA synthesis and mitosis in both cell lines.

### Assay multiplexing

The standard filter set of the Operetta allows the simultaneous determination of four different markers. However, as the emission filters are relatively broad care must be taken when determining which channels to place the various components to be detected in to ensure bleed through does not obscure the detection of the marker in the higher wavelength channel. The SpectraViewer tool from LifeTechnologies (http://www.lifetechnologies.com/uk/en/home/life-science/cell-analysis/labeling-chemistry/fluorescence-spectraviewer.html) is an extremely useful tool for evaluating this. For example, EdU labelling is extremely bright and will result in spill over from the FITC (AF488) channel into the TRITC (AF546) channel. If combined with an antibody that produces a weak signal, for example BrdU, the specificity is lost due to bleed through from the AF488 channel. In such instances, placing the EdU detection in the AF647 channel and the weakest signal in the AF488 channel would be the most advantageous.

Understanding the effects of small molecule inhibitors or biologics on the cell cycle is important for understanding the mechanism of action of drugs especially those being developed as anti-cancer therapeutics. Likewise, the early detection of compounds with adverse effects on the cell cycle has the potential to act as a flag for downstream toxicity. Flow cytometry has remained the gold standard for cell cycle analysis but is laborious and requires trypsinisation of adherent cells which may influence results. High-content analysis is a technique that is readily amenable to cell cycle analysis as it allows the quantification of single cells based on multiple phenotypes. The separation and analysis only of single cells (and not cell clumps) is critical for the accurate determination of cell cycle distribution. The machine-learning algorithm PhenoLOGIC within the Harmony software proved a powerful tool in discriminating single cells from clumps based on a range of morphological and intensity properties. Single cell discrimination was achieved in a range of cell types and importantly following a variety of drug treatments. Accurate cell cycle blocks induced by a range of agents such as M-phase arrest induced by paclitaxel, G2 arrest by etoposide or endoreduplication by the aurora kinase inhibitor VX-680 [16] were readily observed.

An advantage of the 3-colour method utilising DNA content, EdU incorporation and pHH3 (S10) expression with a mouse or rabbit interchangeable pHH3 (S10) antibody is that it can be readily multiplexed with a whole range of additional markers. Using this method, the fraction of quiescent (G0) cells following serum starvation could be readily detected using an anti-Ki67 antibody. Likewise, an analysis of the cell cycle distribution of DNA damage induced by cytotoxic chemotherapeutic drugs such as gemcitabine, camptothecin or etoposide was achievable by multiplexing with a marker of DNA double-strand breaks (γH2AX). Only small increases in cells with a sub-G1 DNA content was observed following treatment with high doses of these agents. However, the process of fixation with formaldehyde has been suggested to prevent the release of small DNA fragments. A distinct advantage of high-content is the ability to reanalyse the images for additional phenotype changes. The fraction of cells with clear nuclear morphological abnormalities (for example change in shape, fragmentation or condensation) could be subsequently determined as an indicator of cell death.

In summary, multiparametric analysis of DNA content, EdU incorporation and pHH3 (S10) expression using the Operetta high-content instrument coupled with the PhenoLOGIC machine learning algorithm in Harmony software is a low cost, rapid and high-throughput method for the accurate determination of cell cycle distribution in adherent cell cultures.

## Supporting Information

S1 Fig Example scatter plot of nuclear area versus nuclear width. The nuclei of HT29 cells was stained with Hoechst 33342 and then segmented with Harmony software using Find Nuclei Method B. Nuclear area and width were calculated in Harmony Software using the building block [Calculate Morphology Properties], Method: Standard.
(TIF)
