# Supplementary figures and images for: Multiparametric Cell Cycle Analysis Using the Operetta High-Content Imager and Harmony Software with PhenoLOGIC
Source: PLoS One. 2015 Jul 28;10(7):e0134306. doi: 10.1371/journal.pone.0134306 (PMC4517780; doi:10.1371/journal.pone.0134306)

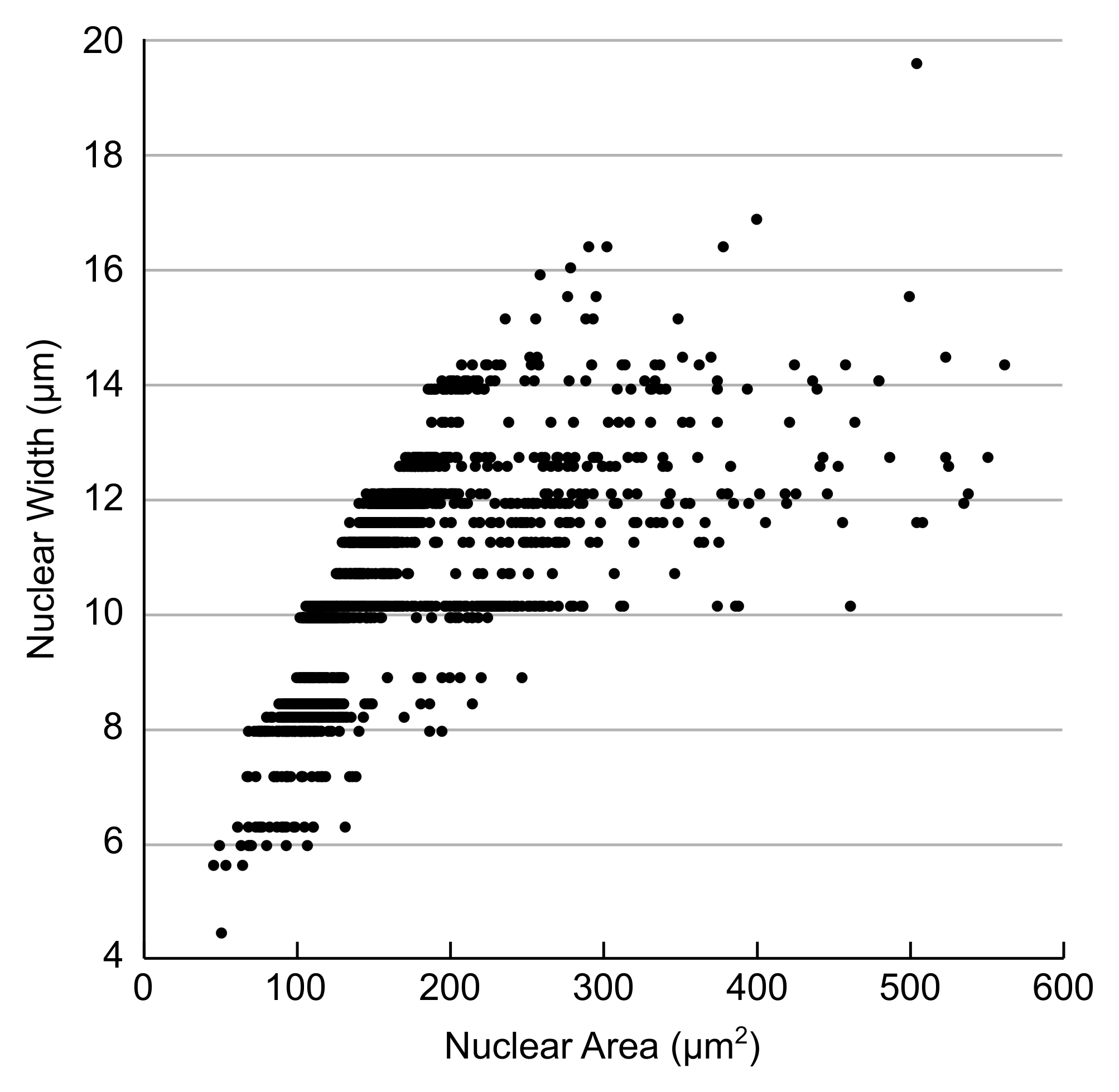

Supplement: S1 Fig — The nuclei of HT29 cells was stained with Hoechst 33342 and then segmented with Harmony software using Find Nuclei Method B. Nuclear area and width were calculated in Harmony Software using the building block [Calculate Morphology Properties], Method: Standard. (TIF) [file pone.0134306.s001.tif]
